# Cortical activation elicited by unrecognized stimuli

**DOI:** 10.1186/1744-9081-2-17

**Published:** 2006-05-16

**Authors:** Rajendra D Badgaiyan

**Affiliations:** 1 Department of Radiology Harvard Medical School; and Massachusetts General Hospital, Boston, MA 02114, USA

## Abstract

**Background:**

It is unclear whether a stimulus that cannot be recognized consciously, could elicit a well-processed cognitive response.

**Methods:**

We used functional imaging to examine the pattern of cortical activation elicited by unrecognized stimuli during memory processing. Subjects were given a recognition task using recognizable and non-recognizable subliminal stimuli.

**Results:**

Unrecognized stimuli activated the cortical areas that are associated with retrieval attempt (left prefrontal), and novelty detection (left hippocampus). This indicates that the stimuli that were not consciously recognized, activated neural network associated with aspects of explicit memory processing.

**Conclusion:**

Results suggest that conscious recognition of stimuli is not necessary for activation of cognitive processing.

## Background

Experiments have demonstrated that subliminal stimuli elicit a variety of cognitive and behavioral responses [1,2]. These stimuli, when used as cues, facilitate identification of primed stimuli [3,4], and improve the speed and accuracy of responses in numerical processing and lexical decision tasks [5-7]. It has been argued that these effects do not necessarily indicate that subliminal stimuli are processed cognitively. The responses elicited by these stimuli could be due to conditioned association of orthographic features of stimuli with the motor response [8-10].

A stimulus driven sensory-motor association could enable subjects to learn to associate non-semantic visual features of the stimulus with a response if stimulus is presented repeatedly [10]. Since most of the experiments on subliminal stimuli have used a limited number of stimuli [e.g., [5,6]], it is difficult to exclude the possibility that responses in these experiments were driven by sensory-motor association. Thus, these results do not provide compelling evidence to suggest that the effects of unrecognized stimuli are driven by cognitive processing, and not by subsemantic sensory-motor association.

Study of the pattern of cortical activation elicited by unrecognized stimuli during performance of a cognitive task could provide additional useful information to help us understand whether these stimuli are cognitively processed. Activation of relevant cortical areas could indicate initiation of cognitive activity, at least at the cortical level. The evidence suggests that the subliminal stimuli become recognizable if they are primed [3,6,7]. In the present experiment this evidence was exploited to understand the pattern of cortical activation elicited by unrecognized stimuli in an explicit memory task.

## Materials and methods

**Figure 1:**
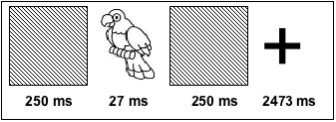
The sequence of stimulus presentation.

The experiment was conducted on 10 right-handed healthy volunteers (mean age 20 years; female 6). At the study stage a series of 120 achromatic line drawings (pictures) of common objects, animals and plants were presented supraliminally for 3 sec each on a computer monitor (refresh rate 75 Hz). Subjects were asked to remember the pictures and make a like/dislike judgment. At the test stage studied (primed condition) and nonstudied (unprimed condition) pictures were shown for 27 msec (ISI 3 sec). These pictures were masked (forward and backward) by neutral patterns, presented for ~250 msec. Subjects were asked to indicate whether a stimulus was studied, nonstudied, or not recognizable, by pressing one of the three keys of a button box and right index, middle, and ring fingers. Each test block had 120 pictures (60 primed and 60 unprimed), which were mixed pseudo-randomly. To allow for the delay in hemodynamic response due to task change, either 4 or 8 primed or unprimed stimuli were presented consecutively. There were two study blocks, each of which was followed by two test blocks. Thus there were a total of four test blocks. Volunteers were not scanned during the study, but they remained in the scanner during the study phase. In the baseline (look-only) condition 120 novel pictures were presented between the neutral patterned masks for 27 msec. The pictures, and the test conditions were counterbalanced across subjects. The ISI was 3000 ms and a cross mark was presented between the stimuli. Subjects were asked to look at the cross mark. There was a break of 2 min between each block. During the break, a fixation cross mark was shown at the center of the monitor. The sequence of stimulus presentation is shown in Figure 1.

Images were acquired using a 1.5 tesla GE Signa scanner, T1 weighted structural images (spin echo, TR 500 msec, TE 16 msec, FOV 20 cm) and T2* weighted blood oxygenation level-dependent (BOLD) images (ISGR, 28 slices, 5 mm thick, 0 skip) were acquired in the coronal plane with echoplanar imaging (gradient echo sequence; TR = 6000 msec, TE = 40, Flip angle = 90°, FOV = 20 cm, 3.125 × 3.125 × 5 mm in-plane resolution, 64 × 64 matrix). Images were motion corrected using an automated image registration algorithm and the magnetic resonance (MR) signal intensities obtained during the test conditions (primed, unprimed and look-only) were compared using pixel-wise analysis of variance. Data obtained from all of the 10 subjects were merged and a random effect model was implemented to determine variance between conditions and across subjects. A 10 × 3 (subjects × conditions) analysis of variance with contiguity threshold of five contiguous pixels was performed on the merged data. The resulting *F*-maps were aligned to the anatomical images and then registered in stereotactic space using AFNI software [11]. A post hoc scan-by-scan analysis was performed on brain regions identified as having significant magnetic resonance (MR) signal change with the omnibus ANOVA. In the primed condition, the scans that were associated with either unrecognized or incorrect responses were not included in the analysis. Similarly, in the unprimed condition, only the scans associated with unrecognized response were included. Each 6-sec scan consisted of two trials, so the MR signal intensity reflected the signal associated with two of either primed or unprimed trials. All the scans associated with the primed and unprimed trials were separated and considered as separate block for further analyses.

## Results

Subjects recognized 76.8% of stimuli presented in the primed condition and correctly recalled 54.6% of these pictures (correct hits 54.6%, misses 22.2%). Stimuli in the unprimed group were generally not recognizable (94.6%). Of the stimuli that were reported as recognized (5.4%), there were 94% correct hits (stated that they were not studied) and 6% false alarm. Mean response time for the primed pictures (975 ± 42) was significantly shorter (p < 0.01) than that for the unprimed pictures (1201 ± 54 msec).

**Figure 2:**
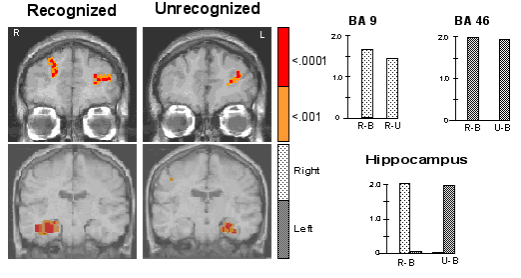
The figure shows increased activation in the prefrontal and hippocampal regions during explicit retrieval elicited by subliminally presented primed (Recognized) and unprimed (Unrecognized) stimuli. A majority of stimuli in the primed condition were recognized by the volunteers, while in the unprimed condition, most of the stimuli were unrecognized. The prefrontal activity was unilateral (left middle frontal gyrus) in the unprimed (Unrecognized) condition, and bilateral (left middle frontal and right superior frontal gyri) in the primed (Recognized) condition. Primed stimuli activated the right hippocampus while unprimed stimuli activated the left hippocampus. Histograms show percent change in MR signal intensity in different contrast conditions in the areas of interest: R = Recognized (primed) picture; U = Unrecognized (unprimed) picture; B = Baseline condition; BA = Brodmann's area.

As compared to the baseline, increased intensity of MR signal was observed in the prefrontal and hippocampal areas when either primed or unprimed pictures were presented (Figure 2). In addition, increased intensity was observed in the left primary motor cortex in both, primed (Talairach coordinates -54, -8, 44; p < .0001) and unprimed conditions (Talairach coordinates -50, -6, 48; p < .0001).

Primed stimuli elicited significantly greater MR signal intensity in the left middle (BA 46; Talairach coordinates, -31, 45, 21; p < .0001), and right superior frontal (BA 9; Talairach coordinates (x, y, z), 22, 45, 32; p < .0001) gyri while unprimed stimuli activated only the left middle frontal gyrus. Thus, the left middle frontal gyrus was activated in both conditions, but activation in the right superior frontal gyrus was observed only during presentation of primed pictures. The pattern of hippocampal activation was also different for the primed and unprimed stimuli. It was lateralized on the right (Talairach coordinates, 32,18,12; p < .0001) in the primed, and on the left (Talairach coordinates, 30,18,14; p < .0001) in unprimed condition. Since these activations were relative to the baseline condition in which subjects did not make any motor response, activations observed in the primed and unprimed conditions includes those elicited by execution of motor response. These activations however should be similar in the two conditions. Thus the two areas (BA 4 and 46) where activations were observed in both conditions could potentially be due to motor activations. Since BA 46 is generally not associated with motor processing, and a number of studies have implicated this area with mnemonic processing [12,14,15], in this discussion only BA 4 activation is assumed to be due to motor activation.

## Discussion

An interesting finding of this experiment was the observation that unprimed pictures that were not recognized consciously, elicited increased activation in the left prefrontal and hippocampal areas. Since both of these activations are associated with cue related explicit retrieval [12-14], the observation suggests that the cues that were not recognized consciously, might have initiated cognitive processing associated with retrieval attempt and recognition.

Increased activation in the left prefrontal cortex has been reported in tasks that require subjects to match a cue with the primed item [12,14,15]. Since the activation is observed even when a cue fails to retrieve the primed stimulus, it is generally associated with retrieval attempt, rather than the retrieval itself [12,14,15]. The observation of left prefrontal activation in both, primed and unprimed conditions indicates that not only the primed stimuli that were consciously recognized, but also, unprimed stimuli that were not consciously recognized, elicited retrieval attempt.

There was no significant change in signal intensity of the right prefrontal cortex in unprimed condition. This observation is in agreement with the findings of earlier neuroimaging studies that have associated right prefrontal activation with retrieval success [12,14,15], and post retrieval monitoring [16]. Increased activation in the right prefrontal cortex is observed when a cue successfully retrieves a studied item [12,14-16]. Since unprimed stimuli did not elicit successful retrieval, right prefrontal activation was not expected. Primed stimuli, on the other hand, did successfully retrieve studied items and also activated right prefrontal cortex.

Another area where increased activation was observed (in both primed and unprimed conditions) is the hippocampus. The hippocampus plays a critical role in binding multiple representations of a stimulus. The binding facilitates understanding of the semantic properties of a stimulus [17]. Since hippocampal activation was elicited by both, primed and unprimed stimuli, it appears that, along with the primed, unprimed pictures initiated 'binding' and semantic processing. The activation in unprimed condition however was observed only in the left hippocampus, which is associated with 'novelty detection' process [14,18]. It has been suggested that during explicit retrieval novel stimuli initiate semantic search of the pool of 'general knowledge' to facilitate semantic identification of novel stimuli [19]. Since identification of a stimulus as novel requires recognition, and unsuccessful match with studied items, it appears that the unprimed stimuli were cognitively processed to the level of semantic recognition. It is possible that these stimuli were 'recognized' at nonconscious level, but the recognition did not evoked reportable conscious awareness [20].

The primed stimuli activated the right hippocampus, which is involved in the retrieval of studied items [12-14]. Since stimuli were retrieved successfully in the primed condition, the observation is consistent with the findings of previous studies in which supraliminal cues were used. This is particularly significant because the trials in which there were incorrect responses were not used to analyze data in the primed condition.

The most significant observation of this experiment concerns the cortical activations elicited by unrecognized stimuli (unprimed condition). Increased activation in the prefrontal and hippocampal areas elicited by these stimuli indicate that the processing associated with explicit retrieval might have been initiated. It appears that under similar conditions, these stimuli activate the same cortical area that process consciously recognized stimuli. Further, the results do not support the suggestion that effects of unrecognized stimuli are based on the sensory-motor conditioning [10]. In this experiment stimuli were shown subliminally only once, and a single exposure is unlikely to establish sensory-motor associations. Activation of the cortical areas associated with the novelty detection, and retrieval attempt also indicates that consciously unrecognized stimuli were semantically processed and they might have been 'recognized' nonconsciously. A stimulus cannot be identified as novel, and it cannot initiate retrieval attempt unless it is 'recognized' and semantically processed. The interpretation of these results however needs cautious approach because in this experiment cortical activation is taken as empirical indicator for cognitive processing, irrespective of the nature of the phenomenal experience. This study therefore needs to be followed up by more comprehensive experiments to elucidate elicitation of phenomenal experience more explicitly. It is also important to examine whether the processes, such as associative-binding, and response criterion setting, have contributed to the prefrontal activation elicited by unrecognized stimuli.

## Conclusion

The results indicate that during cognitive activation, stimuli that are not consciously perceived, elicit cortical activations that are consistent with cognitive processing. It suggests that conscious recognition of stimuli is not necessary for initiation of cognitive processing.
